# Dietary Supplementation with Oleum Cinnamomi Improves Intestinal Functions in Piglets

**DOI:** 10.3390/ijms19051284

**Published:** 2018-04-25

**Authors:** Dan Yi, Qiuhong Fang, Yongqing Hou, Lei Wang, Haiwang Xu, Tao Wu, Joshua Gong, Guoyao Wu

**Affiliations:** 1 Hubei Collaborative Innovation Center for Animal Nutrition and Feed Safety, Hubei Key Laboratory of Animal Nutrition and Feed Science, Wuhan Polytechnic University, Wuhan 430023, China; yidan810204@whpu.edu.cn (D.Y.); fangqiuhong@whpu.edu.cn (Q.F.); wanglei@whpu.edu.cn (L.W.); xuhaiwang@whpu.edu.cn (H.X.); wutao@whpu.edu.cn (T.W.); g-wu@exchange.tamu.edu (G.W.); 2 Guelph Research and Development Centre, Agriculture and Agri-Food Canada, Guelph, ON N1G 5C9, Canada; joshua.gong@agr.gc.ca; 3 Department of Animal Science, Texas A&M University, College Station, TX 77843, USA

**Keywords:** oleum cinnamomi, growth performance, intestinal function, piglets

## Abstract

The present study was to determine the efficacy of dietary supplementation with oleum cinnamomi (OCM) on growth performance and intestinal functions in piglets. Sixteen piglets (24-day-old) were randomly assigned to the control or OCM groups. Piglets in the control group were fed a basal diet, whereas piglets in the OCM group were fed the basal diet supplemented with 50 mg/kg OCM. On day 20 of the trial, blood samples and intestinal tissues were obtained from piglets. Compared with the control group, dietary OCM supplementation increased (*p* < 0.05) average daily feed intake, plasma insulin levels, villus width and villous surface area in the duodenum and jejunum, DNA levels and RNA/DNA ratios in the ileum, the abundance of *Enterococcus* genus and *Lactobacillus* genus in caecum digesta, mRNA levels for epithelial growth factor receptor (EGFR), Ras, extracellular signal-regulated kinase 1/2 (Erk1/2), b-cell lymphoma-extra large (Bcl-xL), villin, junctional adhesion molecule A (JAM-A), myxovirus resistance (MX) 1, MX2 and regenerating islet-derived protein 3 gamma (REG3G), and protein abundances of Ras and claudin-1, but decreased (*p* < 0.05) diarrhoea incidence; the abundances of *Enterobacteriaceae* family, *Enterococcus* genus, *Lactobacillus* genus, *Bifidobacterium* genus, and *Clostrium coccoides* in the colon digesta, and AMP-activated protein kinase (AMPK) mRNA levels and caspase-3 protein abundance in the jejunal mucosa of piglets. Taken together, these data indicate that dietary OCM supplementation modulates intestinal microbiota and improves intestinal function in weanling pigs. OCM is an effective feed additive and alternative to feed antibiotics for improving intestinal health in swine.

## 1. Introduction

With an increasing concern about the negative impact of certain antibiotic use aspects on public health, many countries are gradually banning the use of antibiotics in animal production. Thus, searching for potential alternatives to antibiotics is pivotal for sustainable development of animal production. To date, many research reports have been focused on the plant extracts as they are natural and abundant with strong antibacterial, antifungal and antiviral activities [1,2]. These phytochemicals can disturb microbial cell structures, increase bacterial cell permeability, and consequently result in bacterial death [2]. Essential oils, one kind of the plant extracts, are commonly obtained by steam distillation and have been used in food preservation, pharmaceutical therapies, alternative medicine, and natural therapies for many years [3].

Oleum cinnamomi (OCM) a type of essential oil commonly used in the food industry because of its special aroma [4]. Previous studies have shown the antibacterial activity of OCM that was extracted from both the bark and leaves of *Cinnamomum burmannii* and *Cinnamomun osmophloeum* [3,4]. The predominant bioactive compound in OCM was reported to be cinnamaldehyde, a potent inhibitor of bacteria, yeast, and filamentous molds [5]. The underlying mechanisms through which cinnamaldehyde exerts the inhibitory effects were possibly via inhibition of microbial ATPase activity and cell wall biosynthesis, and changes in bacterial membrane structure and integrity [5]. Cinnamaldehyde was also developed for medicinal purposes, including the use as anti-inflammatory, anti-emetic and analgesic curatives [6]. There were virtually no side effects reported in cinnamaldehyde application. Gowder [7] found that the acute toxicity of cinnamaldehyde is low, with lethal dose 50% (LD_50_) values ranging from 0.6 to more than 2 g per kg of body weight (BW) in various animal species. Specifically, the oral LD_50_ values of cinnamaldehyde were 1.16 g/kg BW and 2.22 g/kg BW for pigs and rats, respectively [8].

Collectively, there is a paucity of literature on the application of OCM in animal production, although cinnamaldehyde has been extensively studied in human medicine. Our previous study has reported that dietary supplementation with 50 mg/kg OCM attenuated the intestinal injury induced by lipopolysaccharide in piglets [9]. To extend the findings of our previous work, the present study was carried out to determine the effects of OCM on intestinal gene expression and function in piglets.

## 2. Results

### 2.1. Growth Performance and Diarrhoea Incidence

Compared with the control group, dietary supplementation with 50 mg/kg OCM increased (*p* < 0.05) the average daily feed intake (ADFI) of pigs by 13.6%, and decreased (*p* < 0.05) the diarrhoea incidence of piglets by 37.5% (Table 1). Pigs in the OCM group had a numerically 18.5% greater average daily gain (ADG) than that in the control, but the difference was statistically nonsignificant due to the small number of animals per treatment group.

### 2.2. Concentrations of Hormones, IGF-1 and PGE_2_ and in Plasma 

Dietary OCM increased (*p* < 0.05) the concentration of plasma insulin by 55.9% in comparison with the control group (Table 2). However, there were no differences between the control and OCM groups regarding the levels of cortisol, insulin-like growth factor 1 (IGF-1), and PGE_2_ in plasma.

### 2.3. Intestinal Histology

Effects of OCM on intestinal histology are shown in Table 3. Dietary OCM supplementation did not affect (*p* > 0.05) intestinal villus height, as well as crypt depth and villus height/crypt depth. However, pigs receiving the OCM diet exhibited higher (*p* < 0.05) villus width and villous surface area in the duodenum and jejunum than those receiving the basal diet.

### 2.4. DNA Levels, RNA/DNA, and Protein/DNA in the Intestinal Mucosa

In comparison with the control group, dietary OCM supplementation increased (*p* < 0.05) DNA levels (+96.2%) and the RNA/DNA ratio (+73.3%) in the ileum of piglets (Table 4).

### 2.5. Bacterial Populations in the Caecum and Colon Digesta

**Figure 1:**
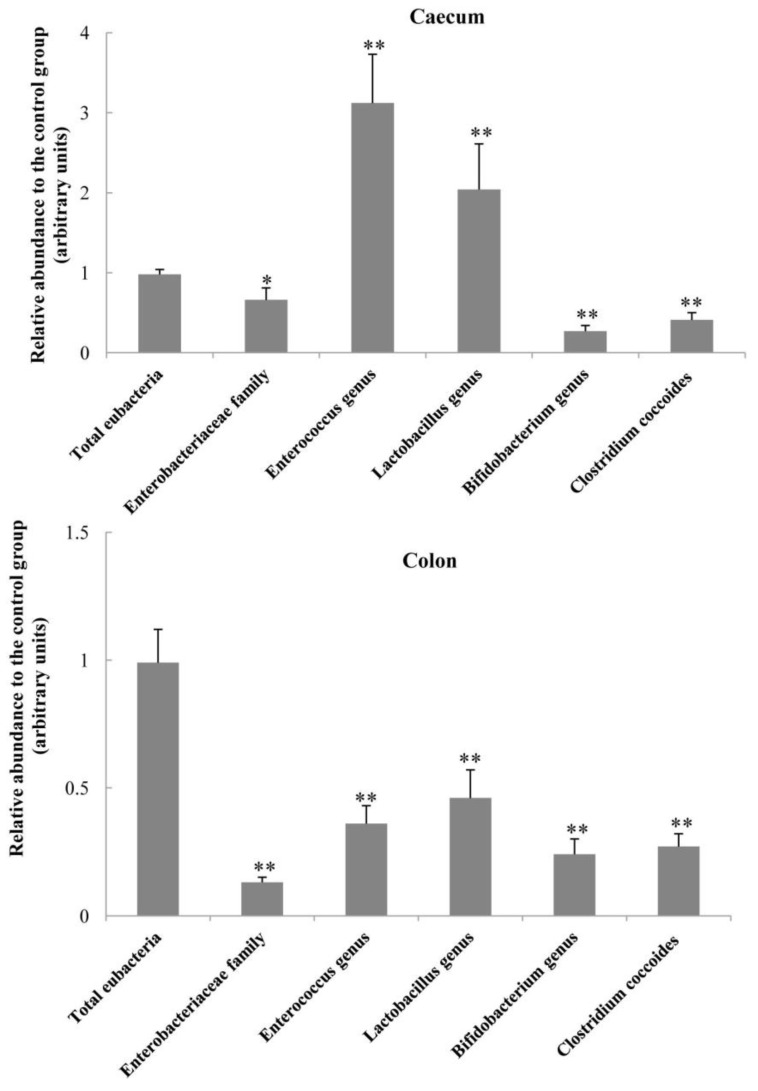
The relative abundance of bacteria in the colon and caecum of piglets. Dietary supplementation with 50 mg/kg oleum cinnamomi (OCM) decreased the abundance of Enterobacterium, *Enterococcus*, *Lactobacillus*, *Bifidobacterium,* and *Clostridium* in the colon, but increased the abundance of *Enterococcus* and *Lactobacillus* in the caecum. DNA levels in the control group were regarded as 1. Data are means ± SD, *n* = 8. * *p* < 0.05, ** *p* < 0.01.

Effects of dietary OCM supplementation on the relative abundance of bacteria in the caecum and colon digesta are illustrated in Figure 1. In the colon, dietary OCM supplementation decreased (*p* < 0.01) the abundance of *Enterobacteriaceae* family (−87%), *Enterococcus* genus (−64%), *Lactobacillus* genus (−54%), *Bifidobacterium* genus (−76%), and *Clostridium coccoides* (−73%), compared with the control group. In the caecum, OCM supplementation increased (*p* < 0.01) the abundance of *Enterococcus* genus (+212%) and *Lactobacillus* genus (+104%), but reduced (*p* < 0.01) the abundance of *Enterobacteriaceae* family (−34%), *Clostridium coccoides* (−59%), and *Bifidobacterium* genus (−73%) in comparison with the control group (Figure 1). In addition, dietary OCM supplementation did not affect the abundance of total bacteria in the caecum and colon digesta.

### 2.6. Gene Expression in the Jejunal Mucosa

**Figure 2:**
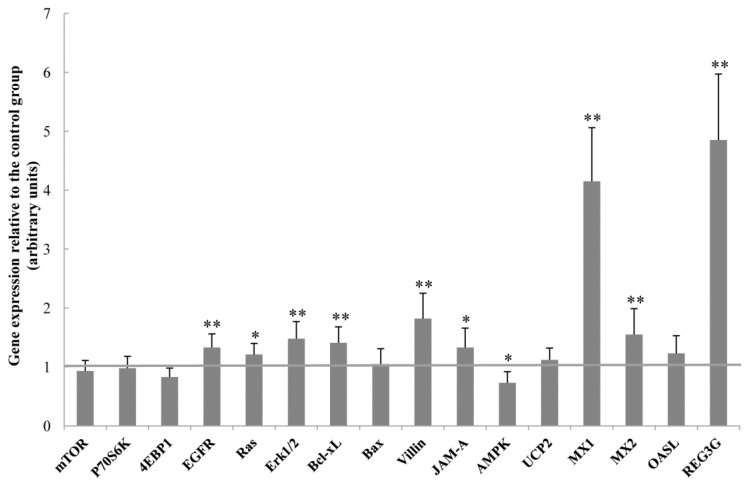
Gene expression in the jejunum of piglets. Dietary supplementation with 50 mg/kg oleum cinnamomi (OCM) up-regulated the expression of EGFR, Ras, Erk1/2, Bcl-xL, villin, JAM-A, MX1, MX2, and REG3G, but down-regulated AMPK expression in the jejunum. mRNA levels in the control group were regarded as 1. Data are means ± SD, *n* = 8. *p < 0.05, **p < 0.01.

Dietary supplementation with OCM affected the gene expression in the jejunum of piglets as shown in Figure 2. Compared with the control group, dietary OCM supplementation increased (*p* < 0.05) the mRNA levels of epithelial growth factor receptor (EGFR, +33%), Ras (+21%), extracellular signal-regulated kinase 1/2 (Erk1/2, +48%), B-cell lymphoma-extra large (Bcl-xL, +41%), villin 1 (+82%), junctional adhesion molecule A (JAM-A, +33%), myxovirus resistance 1 (MX1, +315%), MX2 (+55%), and regenerating islet-derived protein 3 gamma (REG3G, +385%), but reduced (*p* < 0.05) the mRNA level of AMP-activated protein kinase (AMPK, −27%).

### 2.7. Abundance of Caspase-3, Ras, and Claudin-1 Proteins in the Jejunal Mucosa

**Figure 3:**
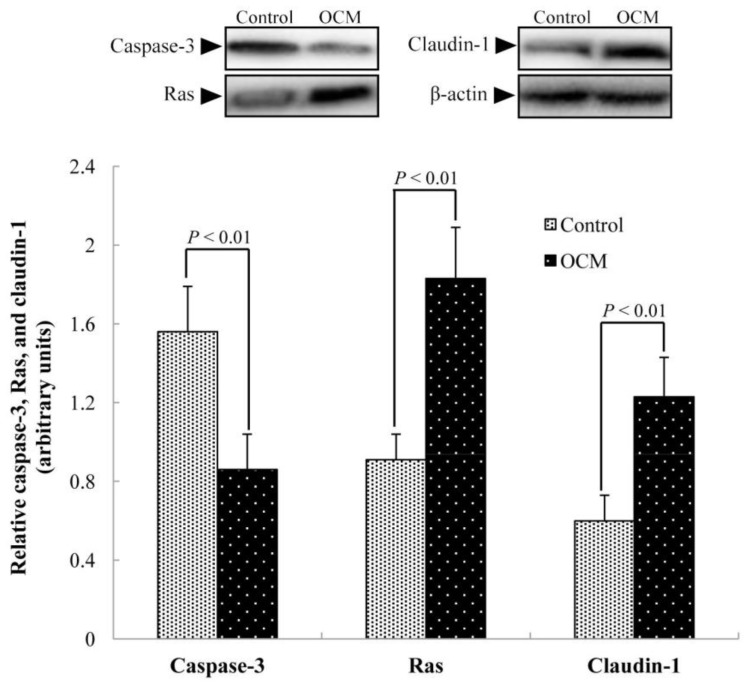
Abundances of caspase-3, claudin-1, and Ras in the jejunal mucosa of piglets. Dietary supplementation with oleum cinnamomi (OCM, 50 mg/kg diet) increased the abundance of Ras and claudin-1 proteins, but decreased the abundance of caspase-3 in the jejunum. Data are means ± SD, *n* = 8.

Compared with the control group, dietary OCM supplementation increased (*p* < 0.01) the abundance of Ras (+101%) and claudin-1 (+105%) proteins, but decreased (*p* < 0.01) the abundance of the caspase-3 protein (−44.9%) in the jejunum (Figure 3).

## 3. Discussion

There is an urgent need to develop antibiotics-free feed, which aims at stopping the spread of antibiotic resistance and can also maintain the current level of animal production. In the present study, as shown by the increased average daily feed intake (ADFI) and reduced diarrhoea incidence, dietary supplementation with 50 mg/kg OCM improved the growth performance of pigs. Similarly, Wang et al. [9] reported that OCM supplementation increased ADFI and average daily gain (ADG), but reduced diarrhoea incidence in pigs that had been challenged with lipopolysaccharide (LPS). The active ingredient of OCM is cinnamaldehyde, which was reported to enhance food intake due to the increased expression of hypothalamic neuropeptide Y in mice [10] and rats [11]. Therefore, dietary OCM may serve as a potential appetite-enhancing feedstuff in weaning piglets. Additionally, concentrations of plasma insulin were increased by the OCM diet, showing that OCM may promote anabolic metabolism in piglets. 

In the present study, dietary supplementation with OCM decreased diarrhoea incidence, indicating that OCM may have beneficial effects on intestinal function. Specifically, OCM supplementation improved the intestinal histology since both villus width and villous surface area were increased in the duodenum and jejunum of pigs in the OCM group. An increase in villous surface area is expected to enhance the digestion and absorption of nutrients [12]. Thus, the OCM diet may augment the bioavailability of dietary nutrients in young pigs. Moreover, dietary OCM supplementation increased ileal DNA concentrations and RNA/DNA ratios, both of which can be used to assess intestinal development [13]. DNA concentration reflects the rate of mitosis for producing new columnar epithelial cells, whereas an RNA/DNA ratio indicates cellular efficiency and a protein/DNA ratio implicates the efficiency of protein synthesis in cells [14]. Therefore, our results demonstrated that dietary OCM supplementation could stimulate the growth of the intestinal mucosa. This notion was substantiated by the results that OCM decreased the intestinal abundance of the caspase-3 protein, which is one of the key components of the apoptotic pathway in the gut [15].

Another important finding in the present study is the alteration of bacterial populations in the intestine by dietary OCM supplementation. The intestinal bacterial mibrobiota contributes to the luminal biological barrier and interacts with the host, thereby affecting the health status of the host [16]. Intestinal diseases are often associated with the changes in enteric bacterial microbiota, such as the increased proliferation of harmful bacteria and the decreased proliferation of beneficial bacteria [17]. In the present study, pigs fed OCM diet exhibited an increase in the populations of *Enterococcus* genus and *Lactobacillus* genus, but a reduction in the abundance of *Enterobacteriaceae* family, *Clostridium coccoides* and *Bifidobacterium* genus in the caecum digesta (Figure 1). However, all of these five bacterial groups were decreased in the colon by OCM treatment, although the total bacterial population was not altered. Our results indicate the differential effects of dietary OCM on intestinal microorganisms. Of note, cinnamaldehyde (the main component of OCM) was reported to be an active inhibitor of bacterial growth via inhibiting microbial ATPase activity, cell wall biosynthesis, and altering membrane structure and integrity [5], whereas OCM had little inhibitory action on *Lactobacillus* and *Bifidobacterium* [1]. Similarly, Nieto-Bobadilla et al. [17] found that oral gavage of CIN-102 (cinnamaldehyde, 86.7% *w*/*w*) significantly reduced the number of luminal and mucosal enterobacteria, and decreased the percentages of bloody stools and diarrhoea in mice. Our results are in line with previous reports, showing that dietary supplementation of OCM can alter the composition of intestinal bacterial microbiota towards the optimal balance of microbiota and consequently lead to the reduction in diarrhoea incidence of piglets. Collectively, considering the reduced diarrhoea incidence and the improvements of intestinal morphology and barrier function, we suggest that the changes in the abundance of selected microorganisms in the caecum and colon in response to dietary OCM supplementation are beneficial for intestinal health in piglets. Further studies are warranted to identify the specific changes in the gut microbiota at the species level.

It is also possible that OCM affects intestinal function by regulating the expression of genes associated with intestinal development, intestinal barrier function, energy metabolism, and anti-viral function. Specifically, OCM up-regulated expression Bcl-xL, an anti-apoptotic protein that promotes cell survival [18], and decreased the abundance of the caspase-3 protein in the jejunum of piglets (Figure 2 and Figure 3), indicating that dietary supplementation with OCM could stimulate intestinal cell growth. This notion was further substantiated by the results that OCM increased the mRNA levels of villin. Villin is a marker of villus cell differentiation [19] and the enhanced expression of villin implies that more villus cells undergo differentiation [20]. Moreover, dietary OCM supplementation increased the mRNA levels of EGFR, Ras, and Erk1/2 in the jejunum (Figure 2). Previous studies indicate that EGF and EGFR are two important mediators for enterocyte proliferation and the regeneration of the mucosal epithelium [21]. Furthermore, the EGFR signaling pathway (the two key downstream molecules are Ras and Erk1/2) was involved in the enhancement of cell proliferation, repair and migration, and stabilization of the internal environment [22]. Given the elevation of the Ras protein abundance by dietary OCM supplementation (Figure 3), we speculate that OCM stimulates enterocyte growth via activating the EGFR signalling pathway. More studies are required to test this hypothesis. 

Regarding the intestinal barrier function, OCM increased junctional adhesion molecule A (JAM-A) mRNA levels and the abundance of the claudin-1 protein in the jejunum. JAM-A is a critical signalling component of the apical junctional complex, which controls the passage of nutrients and solutes across epithelial surfaces [23]. The Claudin family of proteins plays an important role in tight junction formation, thereby influencing intestinal permeability [12]. Thus, OCM may improve intestinal barrier function through regulating intestinal JAM-A and claudin-1 expression. AMPK is a critical regulator of energy metabolism and is activated when the cellular energy level is low [24]. The current study showed that AMPK expression was decreased by dietary OCM supplementation, indicating that OCM may regulate intestinal energy metabolism. Further research is warranted to test this new hypothesis.

The last but important finding of the present work is that dietary OCM supplementation regulated the mRNA levels of antiviral proteins. The myxovirus resistance (MX) proteins are major effector molecules that prevent influenza-infected animals from developing severe phenotypes [25]. MX1 is an important downstream effector of type I interferons and has been reported to possess antiviral activity against a variety of RNA viruses [25]. Our results showed that dietary OCM supplementation increased the mRNA levels for both MX1 and MX2. Therefore, OCM may have the potential to benefit intestinal health by controlling viral infection. Additionally, OCM may consolidate intestinal antimicrobial effect since dietary OCM supplementation dramatically increased the mRNA levels of REG3G, which is expressed predominantly in the small intestine and has been reported to be bactericidal to pathogenic bacteria [26]. Thus, dietary supplementation with OCM can play an important role in improving the intestinal and whole-body health of young pigs. 

In summary, dietary supplementation with 50 mg/kg OCM improved feed intake and and intestinal functions in piglets. The beneficial actions of OCM on intestinal functions include: (1) improvements of intestinal growth and histology (indicated by increased villus height and villous surface area in the duodenum and jejunum; DNA concentrations and RNA/DNA ratios in the ileum; and elevated mRNA levels for EGFR, Ras, Erk1/2, Bcl-xL and villin, and reduced caspase-3 protein abundance); (2) increases in intestinal mucosal barrier function (shown by increased mRNA levels for JAM-A and claudin-1 protein abundance); (3) alterations in the composition of intestinal bacterial microbiota (demonstrated by the reduced population of *Enterococcus*, *Enterobacterium* and *Clostridium* in the colon, and increased abundance of *Enterococcus* and *Lactobacillus* in the caecum); (4) enhancement of antivirus function (indicated by elevated mRNA levels for MX1, MX2, and REG3G); and (5) the reduced incidence of diarrhoea. OCM is a low-cost feed additive and alternative to feed antibiotics for improving intestinal health in weanling pigs.

## 4. Materials and Methods

### 4.1. Animals and Treatments

The animal use protocol for the present study was approved by the Institutional Animal Care and Use Committee at Wuhan Polytechnic University (2014-0514, May 10, 2014). Sixteen crossbred healthy piglets (Durc × Landrace × Yorkshire) were reared by sows and then weaned at 21 ± 1 days of age. After a 3-day adaptation period, piglets (24 ± 1 days of age, average body weight of 5.01 ± 0.50 kg) were housed individually in stainless steel metabolic cages (1.20 × 1.10 m^2^) and maintained at an ambient temperature of 22–25 °C [12]. Piglets had free access to food and drinking water. The corn- and soybean meal-based diet was formulated to meet National Research Council (NRC, 2012)-recommended requirements for all nutrients. All piglets had free access to the basal diet during the 3-day adaptation period. At 24 days of the age (day 0 of the trial), piglets were assigned randomly into one of the two groups: (1) the control group (piglets fed the basal diet) and (2) the OCM group (piglets fed the basal diet supplemented with 50 mg/kg OCM). Each group had eight piglets. OCM was well mixed with the basal diet. To obtain during the 21 days. On day 20 of the trial, blood samples were collected from the anterior vena cava of 12-h fasted pigs into heparinized vacuum tubes and centrifuged (3500× *g* for 10 min at 4 °C approximately isocaloric diets, the control diet was supplemented with 50 mg/kg cornstarch [9]. The experiment lasted for 21 days (from day 0 to 20 days of the trial). Feed consumption, BW, and diarrhoea incidence were recorded) for separating plasma. Plasma samples were then stored at −80 °C until analysis. After collection of blood samples, all piglets were killed under anesthesia with an intravenous injection of pentobarbital sodium (50 mg/kg BW) to obtain the intestinal tissues and the contents.

Oleum cinnamomi was purchased from Sigma-Aldrich Chemicals (St. Louis, MO, USA; Cat. W225800; CAS.8007-80-5). It was extracted from *Cinnamomum cassia* blume by using the steam distillation method. The composition of OCM was: benzaldehyde (0.05%), benzenepropanal (0.37%), *c*innamaldehyde (93.27%), cinnamic acid (0.86%), cinnamyl acetate (0.15%), chlorine compounds (≥1.0%), arsenic (≤3 mg/kg), cadmium (≤1 mg/kg), mercury (≤1 mg/kg), and lead (≤10 mg/kg). The dosage (50 mg/kg OCM) was chosen according to our previous studies indicating that dietary supplementation with 50 mg/kg OCM exerted positive effects on the small intestine in lipopolysaccharide-challenged piglets [9].

### 4.2. Intestinal Sample Collection

The pig abdomen was opened immediately and the whole gastrointestinal tract was immediately exposed [27]. The intestine was dissected free of the mesentery and placed on a chilled stainless steel tray. Segments (5 and 10 cm in length) were obtained from the distal duodenum, mid-jejunum, and mid-ileum [28], respectively. The 5-cm intestinal segments were flushed gently with ice-cold PBS and then placed in a 10% fresh, chilled formalin solution for morphological measurements [12]. On the other hand, the 10-cm segments were opened longitudinally, and contents were carefully flushed with ice-cold PBS. Mucosa was collected by scraping using a sterile glass microscope slide at 4 °C, rapidly frozen in liquid nitrogen, and stored at −80 °C until analysis.

Additionally, to determine the relative microbial amounts by the qPCR method [29], the digesta in the caecum and mid-colon were collected and frozen immediately at −80 °C until further processing [30]. All samples were collected within 15 min after pigs were euthanized.

### 4.3. Plasma Hormones, IGF-1 and PGE_2_

Concentrations of insulin, cortisol, insulin-like growth factor 1 (IGF-1), and prostaglandin E_2_ (PGE_2_) in plasma were analyzed using commercially available ^125^I kits (Beijing North Institute of Biological Technology, Beijing, China) as previously described by Kang et al. [31,32] and Hou et al. [21]. The detection limit was 2 μU/mL for insulin and 1 ng/mL for cortisol. The intra- and inter-assay coefficients of variation were 10% and 15% for insulin, respectively, and were <10% and <15% for cortisol, respectively. Additionally, the detection limits for IGF-1 and PGE_2_ analyses were 21 ng/mL and 0.12 pg/mL, respectively. The coefficients of variation for intra- and inter-assays of IGF-1 were less than 15% and 10%, respectively, and were less than 7.5% and 10.5% for PGE_2_, respectively. 

### 4.4. Intestinal Histology

To determine intestinal histology, three paraformaldehyde-fixed intestinal segements (from duodenum, jejunum, and ileum) were dehydrated and embedded in paraffin. Five-μm sections were cut and then stained with hematoxylin and eosin. Intestinal histology was determined using a light microscope (Leica, Germany) with Leica Application suite image analysis software (Leica, Germany). Only vertically oriented villi and crypts were measured [12]. Histological indices, such as villus height (from the tip of the villi to the villus crypt junction), villus width at half-height, and crypt depth (defined as the depth of the invagination between adjacent villi) were determined from ten adjacent villi [33]. The villus:crypt ratio and villous surface area were calculated. All intestinal histological analysis was done by the same person, who was blinded to the treatments.

### 4.5. Intestinal Mucosal DNA, RNA and Protein

The DNA, RNA and protein in the mucosa were extracted using the TRI REAGENT-RNA/DNA/Protein isolation reagent and their concentrations were determined colorimetrically [12]. DNA was analyzed fluorimetrically using the method of Prasad et al. [34]. RNA was determined by spectrophotometry using a modified Schmidt-Tannhauser method as described by Munro and Fleck [35]. Protein was analyzed according to the method of Lowry et al. [36]. For the measurement of DNA and RNA concentrations, the mucosa was homogenized (T10 basic ULTRA-TURRAX, IKA^®^-Werke GmbH & Co. KG, Neckarsulm, Germany) in a 100-fold volume of ice-cold saline (0.9%) and the homogenate was centrifuged (1,800× *g* for 10 min at 4 °C) to obtain the supernatant fluid for analysis. For the measurement of mucosal protein, mucosal samples (~0.1 g) were homogenized in a 1 mL of ice-cold PBS-EDTA buffer (0.05 M Na_3_PO_4_, 2.0 M NaCl, 2 mM EDTA, pH 7.4) and homogenates were then centrifuged (12,000× *g* for 10 min at 4 °C) to obtain the supernatant fluid for assays.

### 4.6. Microbial DNA Extraction

The bacterial DNA was extracted from colon and caecum digesta and purified using the QIAamp DNA Stool Mini Kit (No. 51504; Qiagen, West Sussex, UK) as described by Castillo et al. [37]. Briefly, each frozen digesta sample (0.3 ~ 0.5 g) was thawed and homogenized in the InhibitEX buffer (Qiagen, West Sussex, UK) and centrifuged to obtain the supernatant fluid. After the addition of proteinase K (Qiagen, West Sussex, UK) to the supernatant fluid, the solution was mixed by vortexing and then centrifuged to collect the supernatant fluid. The latter was mixed with ethanol (96–100%), and DNA was purified by using the QIAamp spin column (Qiagen, West Sussex, UK). Total DNA was quantified using the NanoDrop^®^ ND-1000A UV-VIS spectrophotometer (Thermo Scientific, Wilmington, DE, USA) at an OD of 260 nm, and its purity was assessed by determining the OD260nm/OD280nm ratio. All of the samples had an OD260nm/OD280nm ratio of 1.7 to 1.9. The length of the genomic DNA in each sample was determined using 1% denatured agarose gel electrophoresis. The microbial DNA was stored at −20 °C until qPCR analysis.

### 4.7. QPCR Analyses for Mucosal Gene Expression and Intestinal Bacteria 

Each frozen jejunal mucosal sample (~100 mg) was powdered under liquid nitrogen using a mortar and pestle. The powdered samples were homogenized, and total RNA was isolated using the TRIzol Reagent protocol (Invitrogen, Carlsbad, CA, USA). Total RNA was quantified using the NanoDrop^®^ ND-1000A UV-VIS spectrophotometer (Thermo Scientific, Wilmington, DE, USA) at an OD of 260 nm, and its purity was assessed by determining the OD260/OD280 ratio. All of the samples had an OD260/OD280 ratio above 1.8 corresponding to 90–100% pure nucleic acids [21,38]. Meanwhile, RNA integrity in each sample was determined using 1% denatured agarose gel electrophoresis. RNA was used for RT-PCR analysis when it had a 28 S/18 S rRNA ratio ≥1.8. Total RNA was reverse-transcribed using a PrimeScript^®^ RT reagent kit with gDNA Eraser (Takara, Dalian, China) according to the manufacturer's instruction. cDNA was synthesized and stored at −20 °C until use.

To amplify mucosal cDNA fragments and bacterial DNA, the primer pairs (Table 5) were used for qPCR as previously described by Hou et al. [21] and Ott et al. [16]. To minimize the amplification of potentially contaminating genomic DNA, the primers were designed to span introns and intron-exon boundaries. The qRT-PCR was performed using the SYBR^®^ Premix Ex Taq^TM^ (Takara, Dalian, China) on an Applied Biosystems 7500 Fast Real-Time PCR System (Foster City, CA, USA). The total volume of the PCR reaction system was 50 μL. In brief, the reaction mixture contained 0.2 µM of each primer, 25 µL of SYBR^®^ Premix Ex Taq^TM^ (2×) and 4 µL of cDNA/DNA. All PCR analyses were performed in triplicate on a 96-well real-time PCR plate (Applied Biosystems, Foster City, CA, USA) under the following conditions (two-step amplification): 95 °C for 30 s, followed by 40 cycles of 95 °C for 5 s and 60 °C for 31 s. A subsequent melting curve (95 °C for 15 s, 60 °C for 1 min and 95 °C for 15 s) with continuous fluorescence measurement and final cooling to room temperature was processed. The specificity of the qRT-PCR reactions was assessed by analysing the melting curves of the products and size verification of the amplicons [39]. To ensure the sensitivity and accuracy of the results obtained by qPCR, mucosal samples were normalized internally using simultaneously the average cycle threshold (Ct) of ribosomal protein L4 (RPL4) and glyceraldehyde-3-phosphate dehydrogenase (GAPDH) [40], while microbial samples were normalized using the average Ct of 16S rDNA of eubacteria [29] as a reference in each sample to avoid any artefact of variation in target genes. Results were analysed by 2^−ΔΔCt^ method [41]. 

### 4.8. Protein Immunoblot Analysis 

Protein immunoblot analysis was carried out in accordance with the previously described method [42]. Briefly, frozen jejunal mucosal samples (~100 mg) were powdered and homogenized, in 1 mL lysis buffer, with a homogenizer (T10 basic ULTRA-TURRAX, IKA^®^-Werke GmbH & Co. KG, Germany). After being centrifuged (12,000× *g*, 15 min, 4 °C), the supernatant fluid was aliquoted into micro-centrifuge tubes, to which 2 × sodium dodecyl sulphate (SDS) sample buffer was added in a 1:1 ratio. The samples were boiled and cooled on ice before use for Western blotting. Proteins were separated by electrophoresis on a 10% polyacrylamide gel, and then electrophoretically transferred to a polyvinylidene difluoride (PVDF) membrane. Skim-milk powder in Tris-buffered saline and Tween 20 (TBST) was used to block the membrane for 1 h at 22 °C. Membranes were incubated overnight at 4 °C with one of the primary antibodies: claudin-1 (1:1000, Invitrogen Technology, Grand Island, NY, USA), caspase-3 (1:1000, Cell Signaling Technology, Danvers, MA, USA), Ras (1:1000, Cell Signaling Technology, Danvers, MA, USA), or β-actin (1:5000, Sigma Chemicals, Saint Louis, MO, USA). The membranes were washed with TBST and incubated for 1 h at 22 °C with the anti-rabbit (mouse) immunoglobulin G horseradish peroxidase conjugated secondary antibody (Beijing ZhongShan Golden Bridge Biological Technology Co., LTD, Beijing, China). After being washed with TBST, blots on the membrane were developed using the Enhanced Chemiluminescence Western blotting kit (ECL-plus, Amersham Biosciences, Uppsala, Sweden), visualized and quantified using an imaging system (Alpha Innotech FluorChem FC2, San Leandro, CA, USA). The abundance of caspase-3, claudin-1, and Ras was expressed relatively to β-actin protein.

### 4.9. Statistical Analysis

All experimental data are expressed as means ± SD. The incidence of diarrhoea was analyzed using χ^2^ analysis. Difference of means was determined by the Student’s unpaired *t*-test. Probability values ≤0.05 were taken to indicate significance.

## 5. Conclusions

Dietary supplementation with OCM enhances feed intake, modulates intestinal microbiota, improves intestinal functions, and reduces the incidence of diarrhoea in weanling pigs. OCM is an effective alternative to feed antibiotics for swine. 

## Figures and Tables

**Table 1:** Effects of dietary supplementation with OCM (50 mg/kg diet) on growth performance and diarrhoea incidence of piglets.

Item	Control Group	OCM Group	*p*-Value
ADG (g/d)	260 ± 60.4	308 ± 60.3	0.138
ADFI (g/d)	391 ± 53.9 ^b^	444 ± 33.9 ^a^	0.035
F/G	1.54 ± 0.23	1.42 ± 0.21	0.308
Diarrhoea incidence (%)	9.52 ± 1.29 ^a^	5.95 ± 1.10 ^b^	<0.001

Data are means ± SD, *n* = 8, OCM, oleum cinnamomi; ADG, average daily gain; ADFI, average daily feed intake; F/G, feed/gain ratio. ^a,b^ Means within rows with different superscripts differ (*p* < 0.05).

**Table 2:** Effects of dietary supplementation with OCM (50 mg/kg diet) on the concentrations of hormones, IGF-1 and PGE_2_ in piglets.

Item	Control Group	OCM Group	*p*-Value
Insulin (μU/mL)	5.24 ± 1.07 ^b^	8.16 ± 1.30 ^a^	0.038
Cortisol (ng/mL)	49.3 ± 11.0	42.8 ± 7.96	0.499
IGF-1 (ng/mL)	235 ± 57.4	242 ± 57.9	0.193
PGE_2_ (pg/mL)	29.3 ± 3.09	29.0 ± 2.76	0.813

Data are means ± SD, *n* = 8. OCM, oleum cinnamomi; IGF-1, insulin-like growth factor 1; PGE_2_, prostaglandin E_2_. ^a,b^ Means within rows with different superscripts differ (*p* < 0.05).

**Table 3:** Effects of dietary supplementation with OCM (50 mg/kg diet) on the intestinal histology of piglets.

Item	Control Group	OCM Group	*p*-Value
Villus height (µm)			
Duodenum	246 ± 33.1	249 ± 21.1	0.834
Jejunum	241 ± 37.3	258 ± 18.9	0.281
Ileum	227 ± 44.2	249 ± 15.4	0.090
Crypt depth (µm)			
Duodenum	83.0 ± 13.5	93.5 ± 14.4	0.302
Jejunum	88.4 ± 10.9	94.9 ± 12.4	0.283
Ileum	80.2 ± 14.8	91.9 ± 10.8	0.062
Villus height/crypt depth			
Duodenum	2.98 ± 0.18	2.66 ± 0.23	0.325
Jejunum	2.75 ± 0.31	2.75 ± 0.39	0.906
Ileum	2.85 ± 0.40	2.74 ± 0.36	0.634
Villus width (µm)			
Duodenum	121 ± 12.2 ^b^	147 ± 8.61 ^a^	<0.001
Jejunum	125 ± 12.4 ^b^	149 ± 8.92 ^a^	<0.001
Ileum	104 ± 10.9	116 ± 22.1	0.216
Villous surface area (µm^2^)			
Duodenum	20803 ± 3305 ^b^	25544 ± 2114 ^a^	0.004
Jejunum	20029 ± 3086 ^b^	25729 ± 1617 ^a^	<0.001
Ileum	15419 ± 3252	18593 ± 3644	0.087

Data are means ± SD, *n* = 8. OCM, oleum cinnamomi. ^a,b^ Means within rows with different superscripts differ (*p* < 0.05).

**Table 4:** Effects of dietary supplementation with OCM (50 mg/kg diet) on DNA levels and RNA/DNA and protein/DNA ratios in the intestine of piglets.

Item	Control Group	OCM Group	*p*-Value
DNA (mg/g protein)			
Duodenum	2.49 ± 0.42	2.32 ± 0.33	0.380
Jejunum	2.52 ± 0.49	2.30 ± 0.57	0.605
Ileum	1.84 ± 0.33 ^b^	3.61 ± 0.74 ^a^	<0.001
RNA/DNA ratio			
Duodenum	0.17 ± 0.04	0.16 ± 0.03	0.323
Jejunum	0.16 ± 0.02	0.17 ± 0.04	0.890
Ileum	0.15 ± 0.03 ^b^	0.26 ± 0.02^a^	<0.001
Protein/DNA ratio			
Duodenum	417 ± 71.5	422 ± 96.4	0.918
Jejunum	413 ± 89.2	435 ± 108	0.390
Ileum	533 ± 71.9	555 ± 65.2	0.542

Data are means ± SD, *n* = 8. OCM, oleum cinnamomi. ^a,b^ Means within rows with different superscripts differ (*p* < 0.05).

**Table 5:** Sequences of the primers used for quantitative real-time PCR analysis.

Genes	Forward	Reverse
Bax	TTTCTGACGGCAACTTCAACTG	AGCCACAAAGATGGTCACTGTCT
Bcl-xL	GAAACCCCTAGTGCCATCAA	GGGACGTCAGGTCACTGAAT
Villin	TATTATTGGTGTTCGTGCTA	TCTGGAGGAATAGGATACTAA
JAM-A	AATCAGTGTTCCCTCCTCTGCTAC	ACGGTTGCTCTTGGGCTCT
mTOR	TTGTTGCCCCCTATTGTGAAG	CCTTTCGAGATGGCAATGGA
P70S6K	GGAAACAAGTGGAATAGAGCAGATG	TTGGAAGTGGTGCAGAAGCTT
4EBP1	CCGGAAGTTCCTAATGGAGTGT	GGTTCTGGCTGGCATCTGT
EGFR	GGCCTCCATGCTTTTGAGAA	GACGCTATGTCCAGGCCAA
Ras	AAGAGCGACCTCACCACCA	GCGTTCTTGGCACTCGTCT
Erk1/2	AAGCTCTTGAAGACGCAGCAC	CAGCAGGTTGGAAGGTTTGAG
AMPK	CGACGTGGAGCTGTACTGCTT	CATAGGTCAGGCAGAACTTGC
UCP2	AGGGTCCCCGAGCCTTCT	CAGCTGCTCATAGGTGACAAACA
OASL	GGCACCCCTGTTTTCCTCT	AGCACCGCTTTTGGATGG
MX1	AGTGCGGCTGTTTACCAAG	TTCACAAACCCTGGCAACTC
MX2	CGCATTCTTTCACTCGCATC	CCTCAACCCACCAACTCACA
REG3G	CTGTCTCAGGTCCAAGGTGAAG	CAAGGCATAGCAGTAGGAAGCA
*Enterobacteriaceae* family	CATTGACGTTACCCGCAGAAGAAGC	CTCTACGAGACTCAAGCTTGC
*Enterococcus* genus	CCCTTATTGTTAGTTGCCATCATT	ACTCGTTGTACTTCCCATTGT
*Clostridium coccoides*	AATGACGGTACCTGACTAA	CTTTGAGTTTCATTCTTGCGAA
*Lactobacillus* genus	AGCAGTAGGGAATCTTCCA	CACCGCTACACATGGAG
*Bifidobacterium* genus	TCGCGTC(C/T)GGTGTGAAAG	CCACATCCAGC(A/G)TCCAC
Total eubacteria (16S rRNA)	CGGTCCAGACTCCTACGGG	TTACCGCGGCTGCTGGCAC
RPL4	GAGAAACCGTCGCCGAAT	GCCCACCAGGAGCAAGTT
GAPDH	CGTCCCTGAGACACGATGGT	CCCGATGCGGCCAAAT

## Abbreviations

AMPK	AMP-Activated Protein Kinase
ADFI	Average Daily Feed Intake
ADG	Average Daily Gain
Bax	Bcl-2 Associated X Protein
Bcl-xL	B-Cell Lymphoma-Extra Large
EGFR	Epithelial Growth Factor Receptor
Erk1/2	Extracellular Signal-Regulated Kinase 1/2
GAPDH	Glyceraldehyde-3-Phosphate Dehydrogenase
JAM-A	Junctional Adhesion Molecule A
LPS	Lipopolysaccharide
mTOR	Mammalian Target of Rapamycin
MX1	Myxovirus Resistance 1
OASL	2′-5′-Oligoadenylate Synthetase-Like Protein
OCM	Oleum Cinnamomi
P70S6K	Ribosomal Protein S6 Kinase
REG3G	Regenerating Islet-Derived Protein 3 Gamma
RPL4	Ribosomal Protein L4
UCP2	Uncoupling Protein 2
4EBP1	4E-Binding Protein-1
